# Contact-mediated triclosan delivery for transmission blocking of *Plasmodium* in *Anopheles stephensi*

**DOI:** 10.3389/fmicb.2026.1927723

**Published:** 2026-09-29

**Authors:** Meenakshi Jeena, Gaurav Kumar, Bharti Goyal, Suman Lata, Lipun Kumar Pradhan, Yamini Thakur, Gaganpreet Kaur, Vaishali Verma, Kailash C. Pandey, Shweta Pasi, Jaspreet Kaur

**Affiliations:** 1 Department of Vector Genomics, ICMR-National Institute of Malaria Research (ICMR-NIMR), Dwarka, New Delhi, India; 2 Academy of Scientific and Innovative Research (AcSIR), Ghaziabad, Uttar Pradesh, India; 3 Post Graduate Department of Chemistry, Multani Mal Modi College, Punjabi University, Patiala, Punjab, India; 4 Molecular Biology Division, ICMR-National Institute of Nutrition, Hyderabad, Telangana, India

**Keywords:** *An. stephensi*, contact exposure, bottle assay, *Plasmodium berghei*, transmission blocking, oocyst reduction

## Abstract

**Introduction:**

Extensive and long-term insecticide use has led to the development of resistance in mosquito vectors, underscoring the need for novel insecticidal molecules and integrated, multifaceted strategies that simultaneously target both the vector and the parasite. In line with this, we evaluated the potential of a lipophilic drug triclosan, as a contact-mediated inhibitor to block the development of *Plasmodium* parasite within the malaria vector, *Anopheles stephensi*.

**Methods:**

Molecular docking and simulations identified the potential of triclosan to interact with the ookinete surface proteins of the *Plasmodium* species. Intrinsic toxicity of triclosan was first established on both larval and adult stages of laboratory reared *An. stephensi* through oral feeding and contact exposure (topical application and bottle bio-assay) in terms of survival and/or fecundity and egg hatchability. The transmission blocking potential of triclosan was initially tested *in vitro* using exflagellation assays and ookinete development assays. Finally, studies with *P. berghei* challenged mosquitoes were done to check the *in vivo* transmission blocking activity of triclosan through bottle bioassays.

**Results:**

Docking and simulations showed stable binding of triclosan to *Plasmodium* ookinete surface proteins. Contact exposure of triclosan was non-toxic for adult mosquitoes. However, parasite development was markedly affected. We observed a concentration dependent reduction in exflagellation centers and ookinete formation with increasing concentrations of triclosan compared to the control group. However, at 70 μM concentration, a significant reduction of 87% in exflagellation centers was observed. There was a substantial reduction in ookinete formation; at 50 μM, it was 97.7%, while at 70 μM, the reduction reached 99%. *In vivo*, triclosan exposure resulted in a substantial decrease in oocyst development, with reductions of up to 86% at higher concentrations.

**Conclusion:**

These findings demonstrate triclosan as a contact-mediated anti-*Plasmodium* agent that disrupts parasite development within the mosquito vector, underscoring its potential as a novel vector-targeted transmission blocking intervention against malaria.

## Introduction

*Anopheles stephensi*, a key urban malaria vector, contributes significantly to malaria transmission in India and is invading Africa in recent times. Vector control remains one of the primary malaria control strategies, largely dependent on insecticides. However, insecticide resistance is a significant concern, with *Anopheles* species developing resistance to various insecticides over time. A review by Raghavendra et al. (2022) highlights the growing insecticide resistance among Indian malaria vectors, hindering effective disease control. The current reliance on chemical insecticides is challenged by resistance development, necessitating the development of alternate and complementary strategies (Faizi and Kaur, 2021; Kumar et al., 2022). These include biological control methods (e.g., genetically modified mosquitoes), integrated vector management (IVM), improved surveillance, and the discovery and development of novel insecticidal compounds. Several innovative strategies have emerged which include the use of insecticide mixtures (Oxborough et al., 2015; Alkenani, 2017; Accrombessi et al., 2023), intervention combinations (Moiroux et al., 2025; Kenea et al., 2019), and attractive toxic sugar baits to enhance mosquito control (Kumar et al., 2023; Jeena et al., 2024). Additionally, transmission-blocking interventions which aim to prevent malaria parasite development within mosquitoes, offer a unique strategy to reduce transmission while avoiding the selection pressure imposed by insecticides on mosquitoes (Kumar et al., 2024). Such approaches could help mitigate the impact of insecticide resistance and strengthen malaria control efforts in endemic regions.

The invasion of mosquito midgut epithelial cells by ookinetes and human hepatocytes by sporozoites constitutes critical population bottlenecks in the *Plasmodium* life cycle (Guissou et al., 2023). Targeting specific molecular interactions between the host and parasite that support sexual stage development offers a promising strategy to interrupt transmission from vector to human. *Plasmodium* ookinetes harbor certain surface proteins which are crucial for ookinete survival, penetration of the mosquito midgut epithelium, and subsequent transformation into oocysts. Among the 10–15 different ookinete surface proteins expressed on the plasma membrane of ookinete, P25 and P28 are predominant (Saxena et al., 2007).

P25 and P28 are key ookinete surface proteins that protect the parasite within the mosquito midgut (Tomas et al., 2001). Significantly, vaccines targeting these proteins have been shown to block parasite transmission, reducing oocyst formation by more than 99% (Smith et al., 2014). These proteins are strikingly conserved across *Plasmodium* species that infect humans, birds, and rodents.

Exposure to transmission-blocking agents commonly occurs through oral intake, either by the vertebrate host or the mosquito vector, as exemplified by compounds such as primaquine and atovaquone. Oral administration may be limited by safety concerns, as seen with primaquine, which can cause hemolytic anemia in individuals with G6PD deficiency.

These limitations highlight the need for alternative delivery routes or compounds that can effectively block parasite transmission with minimal host toxicity. Paton et al. (2019) has shown that exposing mosquitoes to glass surfaces coated with an antimalarial compound atovaquone (ATQ) blocks the development of *Plasmodium* oocyst stage specifically from zygote to ookinete and subsequent oocyst stage in the midgut of the mosquito (Paton et al., 2019). The authors reported complete arrest of parasite development in the midgut of treated *Anopheles gambiae* mosquitoes. ATQ being a highly lipophilic molecule was well absorbed into the mosquito body through contact similar to insecticides.

Triclosan having chemical name as [5-chloro-2-(2,4-dichlorophenoxy) phenol], a generally used disinfectant, exhibits potent antiplasmodial activity against *Plasmodium falciparum in vitro* and is effective in a murine *Plasmodium berghei* infection model (Surolia and Surolia, 2001). Studies have demonstrated that triclosan targets the enzyme enoyl ACP reductase, which is an essential enzyme of the *Plasmodium's* fatty acid synthesis pathway (Kapoor et al., 2004). Triclosan demonstrates broad inhibitory activity against dihydrofolate reductases of *P. falciparum* and *P. vivax*, including pyrimethamine-resistant variants (Bilsland et al., 2018). Despite these documented activities, the effects of triclosan on the sexual stages of the parasite remain poorly understood. Physicochemical profile of triclosan with it's Log Partition Coefficient of 5.23, and the topological polar surface area of the drug is 29.46 Å^2^, suggest that triclosan possesses properties conducive to penetration through the lipid-rich mosquito cuticle and subsequent interactions to display transmission blocking activity. Together, these gaps in understanding suggest that triclosan holds considerable promise as a dual-acting antimalarial targeting both asexual and sexual stages of *Plasmodium*. We hypothesized that contact exposure to triclosan enables its penetration through the mosquito cuticle, thereby interfering with the development of the sexual and mosquito stages of *Plasmodium berghei* within *Anopheles stephensi*, resulting in reduced parasite transmission while exerting minimal adverse effects on mosquito survival under the experimental conditions tested. Our initial experiments involved *in silico* analysis to predict the possible interactions between triclosan and ookinete surface proteins. We then assessed the intrinsic toxicity of triclosan on the adult and larval stages of *An. stephensi*. Subsequently, mosquitoes were exposed to various concentrations of triclosan coated on glass bottles and then infected with *Plasmodium berghei* using an infectious blood-meal. Parasite development measured as rate of oocyst formation was assessed quantitatively in the midgut of mosquitoes to determine the impact of triclosan exposure (Figure 1).

**Figure 1:**
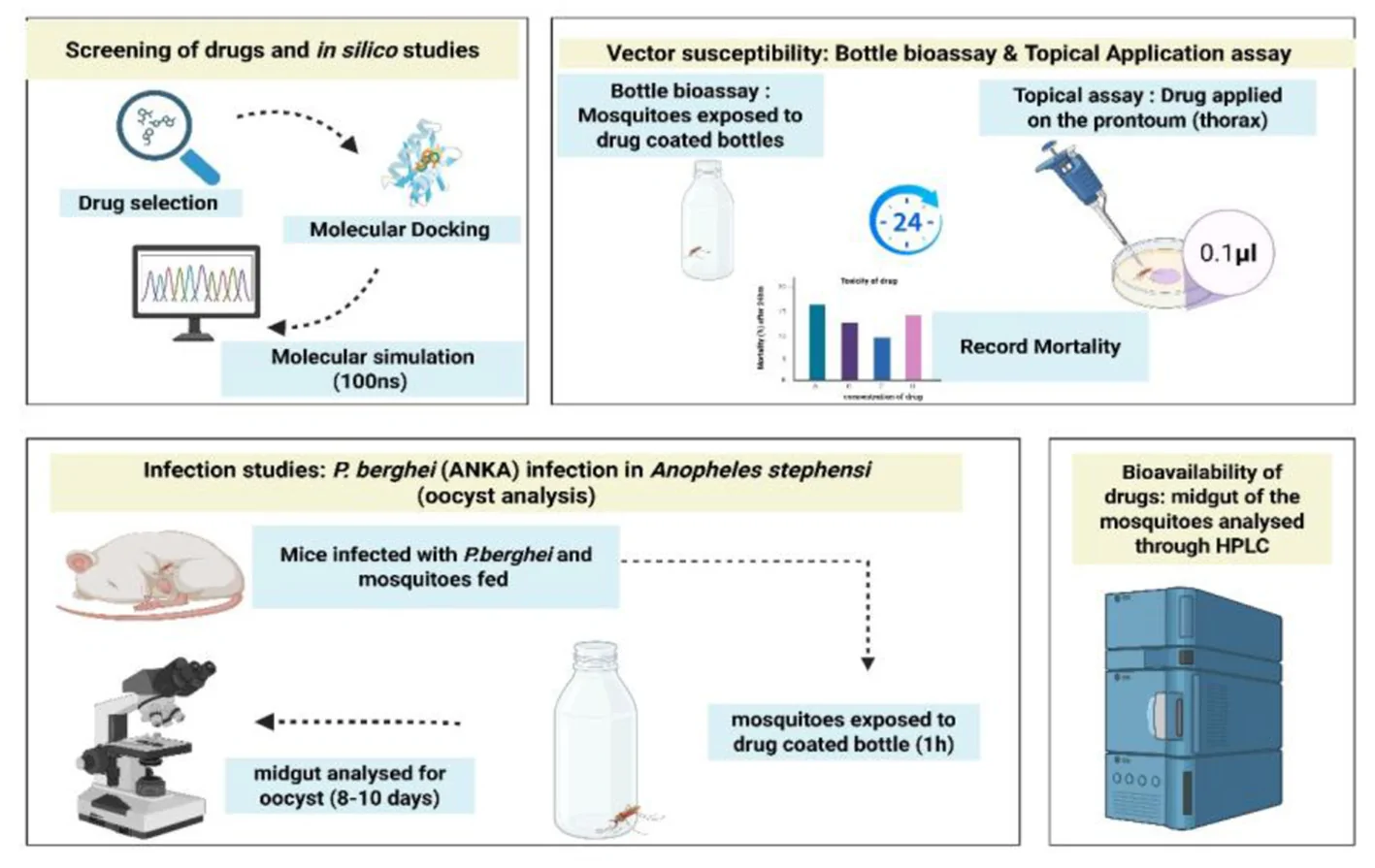
Experimental workflow for the evaluation of triclosan against *Anopheles stephensi* and *Plasmodium berghei* infection.

## Materials and methods

### Docking and simulations

Triclosan was docked against *Plasmodium* ookinete surface proteins to identify the potential interactions between the drug molecule and the target proteins on ookinete surface. Three ookinete surface proteins-Q8IJ96, Q04620, Q71TS7, having the common conserved domain (P28domain, accession no: pfam06247) were selected for docking analysis (Table 1). The alpha fold server was used to predict the structure of all the ookinete proteins. The obtained protein structures were further validated with PROCHECK software.

**Table 1:** The three ookinete surface proteins having common conserved domain that have been analyzed for molecular docking.

Ligand	Ookinete surface proteins	Conserved domain
TRICLOSAN	Q04620, *P. berghei* (Anka) ookinete surface protein	P28 domain
	Q71TS7, *P. berghei* (Anka) ookinete surface protein	
	O8IJ96, *P. falciparum*(3D7) ookinete surface protein	

The key proteins are the P25 and P28 ookinete surface proteins, characterized by conserved EGF domains (Baton and Ranford-Cartwright, 2005). EGF-like domains, which are evolutionarily conserved, are essential for malaria parasite development in the mosquito, playing a key role in both ookinete survival and oocyst formation. Using the FASTA sequences of the respective proteins, conserved domains and corresponding templates were obtained from the NCBI Conserved Domain Database (CDD v3.21).

### Protein model validation

Predicted three-dimensional structures of the target proteins (Q8IJ96, Q04620, and Q71TS7) were retrieved from the AlphaFold Protein Structure Database. Model confidence was assessed using per-residue Predicted Local Distance Difference Test (pLDDT) scores. Mean pLDDT values and confidence distributions (very high, >90; confident, 70–90; low, < 70) were visualized in PyMOL (v2.5, Schrödinger, LLC, New York, USA). Global model quality was evaluated using the QMEANDisCo server (https://swissmodel.expasy.org/qmean). The QMEANDisCo global score, integrating statistical potentials with distance constraint agreement from homologous structures, was used to estimate the overall structural reliability. Structural geometry and stereochemical quality were assessed using the MolProbity web server (https://molprobity.biochem.duke.edu/). Hydrogen atoms were added using reduce with automatic side-chain optimization before analysis. Validation included assessment of clash score, Ramachandran plot statistics, side-chain rotamers, Cβ deviations, and bond geometry, from which the overall MolProbity score was obtained.

### Molecular docking study

The structures of three ookinete surface proteins were retrieved from the UniProt database and prepared using the Protein Preparation Wizard in the Maestro interface (Schrödinger v2023-2) to correct missing hydrogen atoms and valency errors. Structures were energy-minimized in the Macro Model module using the OPLS-2005 force field and the Polak–Ribiere conjugate gradient algorithm, with a convergence gradient of 0.01 kcal/mo. Ligand structures were generated and energy-minimized using MacroModel (Schrödinger, New York, USA), followed by geometrical optimization with Jaguar. Multiple ligand conformations were generated using LigPrep (Schrödinger, New York, USA).

In the absence of co-crystal structures, blind docking was performed to predict potential binding sites using SiteMap. Receptor grids were generated using the Glide module, with an inner grid (12 × 12 × 12 Å) defining the docking region and an outer grid (20 × 20 × 20 Å) accommodating ligand flexibility. Ligand conformers were docked into each predicted site using the Glide XP algorithm, and binding poses were evaluated using the Glide XPScore function. The lowest-energy complexes with favorable docking scores were selected for further analysis.

### MD simulation of docked complexes

Molecular dynamics (MD) simulations of the docked triclosan–ookinete surface protein complexes were performed for 100 ns using the Desmond package (Schrödinger 2023-2, New York, USA). Using System Builder package, the system was built in an orthorhombic box with SPC as water model and for neutralization Na^+^ ions were added keeping 0.15 M of ionic concentration. At first a short run of NVT (10 ns), was done to maintain system relaxation followed by the NPT ensemble with the temperature 300 K with a pressure of 1 bar was considered with a relaxation of 1 ns. OPLS 2005 was used as the force field. The long-range electrostatics interactions were calculated using the particle mesh Ewald method with a cutoff radius of 10 Å. The Martyna–Tuckerman–Klein chain coupling scheme with a isotropic coupling constant of 2.0 ps was used for the pressure control and the Nosé–Hoover chain coupling scheme for the temperature control. The r-RESPA integrator was applied for determining nonbonded forces, with short range forces being recorded at each step while the long-range forces are being recorded over the three steps. At every 4.8 ps interval the trajectories were recorded. The analysis of the ligand and protein interactions were determined by the Simulation Interaction Diagram tool of Desmond package (Schrödinger-2023-2). The structural stability of the three protein-ligand complexes was evaluated by monitoring the Cα RMSD of the protein and the ligand RMSD over the 100 ns molecular dynamics trajectories.

### Mosquitoes

Laboratory-maintained colonies of *Anopheles stephensi* were reared in a dedicated insectary at the ICMR–National Institute of Malaria Research, New Delhi, India, under controlled environmental conditions (27 ± 2 °C; 70 ± 10% relative humidity). Adult female mosquitoes (3–5 days old) were subjected to insecticide susceptibility testing using the WHO tube assay (paper method), revealing 70% resistance to deltamethrin and malathion and complete resistance (100%) to DDT (Indian Council of Medical Research, 2023). After overnight hatching of eggs in deionized water, first-instar larvae (150 per pan) were reared in 500 mL deionized water and fed powdered fish food (Tetra Bit Complete). Pupae were subsequently collected and transferred to adult emergence cages, and the emerging adults were maintained on a 10% glucose solution supplemented with raisins as part of routine colony maintenance.

### Working compound

Compound triclosan in a powdered form (>95% purity, Sigma) was dissolved in ethanol for preparation of stock solution of 1Millimolar. The working concentrations in the range of 10 μM to 500 μM were prepared in acetone for assays.

### Coating of bottle with test drug

After rinsing with distilled water, borosilicate narrow-mouthed bottles (Sigma; total surface area = 0.0268 m^2^) were dried overnight in a hot-air oven at 60–70 °C. The dried bottles were rinsed again with acetone under chemical fume hood and left overnight. The control bottle was coated with acetone (vehicle) and the test bottles were coated with 1 ml solution of respective concentration of triclosan (μg/bottle). The bottles were left overnight for complete drying in chemical fume hood.

### Bottle bioassay

The bottle bioassay was performed as per the methodology detailed in Common Protocol of ICMR (Indian Council of Medical Research, 2023). After coating the bottles with the test compound, 3–5-day-old *An. stephensi* mosquitoes (*n* = 25 per bottle) were introduced. Each experimental setup consisted of two control replicates (acetone only) and four replicates for each concentration of the test drug. After a 1 h exposure period, knockdown was recorded and mosquitoes were transferred to insecticide-free holding cups covered with netting and provided with 10% glucose solution. Holding conditions were maintained at 27 ± 2 °C and 80 ± 10% relative humidity, and mortality was recorded after 24 h. All bioassays were performed in triplicate using independent batches of mosquitoes and bottles, and mortality was calculated as the percentage of dead mosquitoes per replicate. Assays were excluded if control mortality exceeded 20%. Triclosan doses were prepared by dissolving the required amount (mg) in 1 mL solvent per bottle, with final concentrations reported as μg per bottle (Table 2).


$$\begin{array}{r} \text{Test mortality }(\%)=(\text{Number of dead mosquitoes in test/}\\ \text{Total mosquitoes exposed in test})\times 100 \end{array}$$

**Table 2:** Triclosan concentrations used for bottle and topical application bioassays.

Triclosan concentration	Bottle bioassay (μg/bottle)	Topical application assay (ng/mg mosquito)
10 μM	2.81	0.193
50 μM	14.47	0.97
100 μM	28.95	1.93
250 μM	72.38	4.82
500 μM	144.77	9.65
1,000 μM	289.54	19.3

### Topical application assay

These experiments were conducted using laboratory-reared, 3–5-day-old blood-fed female *Anopheles stephensi*. Triclosan solutions of varying concentrations were prepared in acetone (Table 2). Groups of 25 mosquitoes were briefly anesthetized with CO_2_ for 25–45 s and placed on a cold plate maintained at 4 °C. The average body weight (mg) of mosquitoes was determined prior to treatment. Each concentration was tested on a total of 50 mosquitoes, with acetone-treated mosquitoes serving as the control. A volume of 0.1 μL of the respective triclosan solution was topically applied to the pronotum of the thorax using a micropipette. Following treatment, mosquitoes were transferred to holding tubes and maintained for 24 h in a climatic chamber at 27 °C and 80% relative humidity. Mosquito mortality was recorded 24 h after treatment and, when applicable, adjusted using Abbott's formula. Toxicity was expressed as nanograms of compound per milligram of mosquito body weight. Each drug assay was performed in triplicate using independent mosquito batches. The dose for each drug was calculated as nanograms of active ingredient per milligram of live mosquito mass. Experiments were excluded if control mortality exceeded 20%; for control mortality between 5 and 20%, treated mortality was corrected using Abbott's formula. Data was analyzed using GraphPad Prism Software (version 10.4.1), Boston, USA. Triclosan (MW: 289.5 g/mol) was applied topically, with the mean individual mosquito weight determined to be 1.5 mg for groups of 25. Drug doses (ng) corresponding to the tested concentrations (μM) were calculated for a 0.1 μL application volume, normalized to mosquito body weight, and reported as ng/mg.

### Larvae susceptibility test

Larvicidal efficacy of triclosan was assessed against third-instar (L3) larvae of *Anopheles stephensi* by exposing them to graded concentrations of triclosan (10, 100, and 1,000 ppm). Bioassays were performed under controlled laboratory conditions (27 ± 3 °C; 80 ± 3% relative humidity) with three independent replicates per concentration, along with a water-treated negative control. Larval mortality was evaluated at each concentration and expressed as percentage mortality.

### Survival assays for oral feeding of triclosan

Female mosquitoes (*n* = 50) were kept in separate, 30 × 30 × 30 cm clothed cages. They were provided triclosan at concentrations–10, 50, 100, 250, 500, 1,000, and 2,000 μM mixed with 5% sucrose. Two replicates for each triclosan concentration were used for the experiment (50 mosquitoes/replicate). In the survival experiments, sucrose was provided as a positive control. Mosquito mortality was monitored daily until complete mortality was achieved. The assays were conducted in three independent biological cohorts, each consisting of 50 adult female mosquitoes per treatment, with two replicates. Daily counts of dead mosquitoes were recorded for each cohort, and survival percentages were calculated independently. Mean survival values were obtained by averaging across all cohorts.

Survival analysis was performed using Kaplan–Meier estimators to assess daily survivorship at different drug concentrations. Differences in survival between treatment groups and the control (5% sucrose) were evaluated using the Mantel–Cox (log-rank) test. Survivorship was expressed as the percentage of mosquitoes remaining alive on each day relative to day 0.

### Fecundity and hatchability

For the fecundity and hatchability assays, newly emerged mosquitoes were reared on different concentrations of triclosan viz., 10, 500, and 1,000 μM prepared in 5% sucrose. At the end of 3 days, blood meal was offered to the mosquitoes. Following blood feeding, triclosan feeding was continued. Ten female mosquitoes from each experimental group were separated and allowed to oviposit individually in plastic bowls (250 ml) lined with moist paper towel. The number of eggs deposited in different experimental groups were counted under a microscope. The deposited eggs were transferred to plastic cups containing 100 ml of water for hatching. All the first instar larvae hatched from deposited eggs were counted. The same batch of female mosquitoes in different experimental groups were provided a second blood meal to assess the effect of drug feeding on the second gonotrophic cycle; each gonotrophic cycle represents one complete cycle of blood feeding, egg development, and oviposition.

All the experiments were performed in triplicate under optimum conditions in the insectary.

### Fecundity and egg hatchability of *An. stephensi* after exposure to triclosan coated bottles

To assess the effects of the triclosan on fecundity and egg hatchability, adult mosquitoes were first exposed to the triclosan using a standard bottle assay for 1 h at concentration; 4,000 μM. Twenty-four hours post-exposure, mosquitoes were provided a blood meal. For each concentration (n = 25 mosquitoes) were used per replicate. The experiment was conducted in triplicate for each treatment concentration. Fecundity was evaluated by counting the number of eggs laid per female, and egg hatchability was determined by calculating the percentage of eggs that successfully hatched.

### *In vitro* exflagellation and ookinete developmental assay

To quantify male gametocyte exflagellation, 5 μL of tail blood from *Plasmodium berghei*–infected mice was mixed with 20 μL of pre-warmed ookinete medium (RPMI 1640 supplemented with 25 mM HEPES, 20% fetal bovine serum, 10 mM sodium bicarbonate, and 50 mM xanthurenic acid; pH 7.6). Exflagellation centers were enumerated 20 min post-activation by examining 12–15 random fields per sample using a 100 × objective on a compound microscope (Olympus). Triclosan was added at final concentrations of 10, 50, and 70 μM to assess its effect on exflagellation. The *in vitro* triclosan concentrations (10–70 μM) were selected based on previous reports of antiplasmodial activity and preliminary optimization experiments (Surolia and Surolia, 2001). Although ATQ was used as a positive control in the *in vivo* oocyst assay, it was not included in the *in vitro* exflagellation or ookinete assays, as its stage-specific activity has been previously characterized, with no significant inhibitory effect on exflagellation but inhibition of ookinete formation (Fowler et al., 1995; Delves et al., 2012).

Ookinete development assays were conducted using the same blood-to-medium ratio. Cultures were incubated in multi-well plates at 21 °C for 2 h to allow gametogenesis and fertilization. Subsequently, 225 μL of fresh ookinete-medium containing the designated triclosan concentration was added, and cultures were maintained at 20 °C for an additional 21–24 h to facilitate ookinete differentiation. Each treatment was carried out in triplicate.

Ookinete quantification was carried out by removing 62.5 μL of medium from each well, resuspending the blood pellet in the remaining volume, and placing a 10 μL aliquot on a glass slide. Ookinetes were counted in 27 microscopic fields per sample using a 100 × objective (Fabbri et al., 2021).

### *Plasmodium berghei* infection in mice

All animal procedures were approved by and conducted in accordance with the guidelines of the Institutional Animal Ethics Committee (Registration No. 33/GOReBi/S/99/CPCSEA). Four- to six-week-old male and female BALB/c mice were intraperitoneally infected with *Plasmodium berghei* ANKA parasites (Jeena et al., 2025). On day 4 post-infection, blood smears from the tail vein were prepared, stained with 1% Giemsa, and examined under 100 × oil immersion to quantify gametocytes. When gametocytemia reached 2%−5% (typically on days 7–8), mosquitoes that had been starved overnight were allowed to feed on infected mice. Fully engorged mosquitoes were collected after 1 h and, 12 h post-blood meal, transferred into drug- or vehicle-coated bottles for a 1-h exposure. Mosquitoes were exposed to triclosan 12 h after the infectious blood meal. This exposure interval was selected to model post-blood-meal contact exposure of mosquitoes resting on treated surfaces and was based on the experimental strategy reported previously, where contact exposure to an antimalarial compound 12 h after an infectious blood meal effectively inhibited subsequent parasite development (Paton et al., 2019). The present design was therefore intended to determine whether triclosan could exert a post-infection transmission-blocking effect following acquisition of the parasite. Following treatment, mosquitoes were returned to cages and maintained in an insect growth chamber set at 19 °C with 80%−90% relative humidity and a 12:12 h light: dark photoperiod, with continuous access to a 10% sucrose solution (Percival Scientific, USA). Mosquito midguts were dissected between the 8th and 10th day following infectious blood feeding, stained with 0.5% mercurochrome, and examined under light microscopy at 10 × and 40 × magnification to detect oocysts (Jeena et al., 2025).

The triclosan exposure range selected for the bottle assays was 50–4,000 μM. Notably, the higher concentrations used in the bottle bioassay for infection studies (50–4,000 μM) represent the nominal surface dose required for contact exposure. As triclosan must penetrate the mosquito cuticle before reaching the parasite, its effective bioavailability is lower than in direct *in vitro* exposure, necessitating higher applied concentrations to establish a dose–response relationship and estimate the ED_50_. For each drug concentration, both oocyst intensity, defined as the number of oocysts per midgut, and oocyst prevalence, defined as the percentage of mosquitoes harboring oocysts, were quantified. The study protocol received approval from the Institutional Animal Ethics Committee of ICMR–NIMR (Reference No. NIMR/IAEC/2024_1/4) and was conducted in accordance with established international ethical guidelines and regulations.

### Bioavailability of triclosan in the midgut of *Anopheles stephensi* (UPLC)

HPLC-grade acetonitrile (99.8%, qualigens) and water (Milli-Q), were used for chromatographic analysis. All solvents were filtered prior to use. Adult mosquitoes were divided into treatment groups (*n* = 25 per concentration). Mosquitoes were exposed to triclosan at concentrations of 100, 2,000, and 4,000 μM for 1 h. Following exposure, mosquitoes were maintained under controlled laboratory conditions for an additional 4-h holding period to allow systemic distribution. All concentration were analyzed in duplicate and repeated twice. After the holding period, midguts were dissected under a microscope. For each replicate, pooled midguts (*n* = 25) were transferred into 100 μL of acetonitrile. Midgut samples were homogenized thoroughly using a tissue homogenizer. The homogenates were centrifuged at 12,000 rpm for 20 min at 4 °C. The supernatant was collected and filtered through a 0.25 μm syringe filter. The filtered extract was transferred into UPLC vials for analysis.

Quantitative analysis was performed using a Waters Acquity UPLC system equipped with a PDA eλ detector. Column used was Acquity UPLC BEH C18 column (2.1 mm × 50 mm, 1.7 μm particle size). Chromatographic Conditions includes flow rate of 0.3 mL/min with Injection volume of 5 μL, Column temperature was 35 °C, Detection wavelength of 280 nm, run time was 6 min. Triclosan was identified based on retention time and UV absorption spectrum compared with standard reference. A stock solution of triclosan (1 mg/mL) was prepared in acetonitrile. Working standards were prepared by serial dilution to obtain concentrations ranging from 10 to 1,000 ng/mL. Each standard was injected in triplicate. A calibration curve was constructed by plotting peak area vs. concentration. Linearity was evaluated using linear regression analysis. Triclosan concentrations in midgut samples were calculated using the calibration curve equation. Results were expressed as ng triclosan per mosquito midgut.

### Statistical analysis

Data were analyzed using GraphPad Prism (version 8.0.2).The effects of different triclosan concentrations on mosquito mortality in bottle and topical assays were assessed using oneway ANOVA followed by Dunnett's multiple-comparison test. Results are expressed as mean ± SD, with *p*-values indicating statistical significance. For *in vivo* and *in vitro* infection experiments, results are similarly reported as mean ± SD, with statistical significance evaluated relative to the control group. Dose–response relationships for oocyst inhibition were analyzed using nonlinear regression models, and the median effective dose (ED50) for oocyst reduction was estimated using the dose–response function in GraphPad Prism. UPLC bioavailability experiments were performed in duplicate. Data were expressed as mean ± SEM. Statistical significance among treatment groups was analysed using one-way ANOVA. Differences were considered statistically significant at *p* < 0.05.

## Results

### Molecular docking reveals binding of triclosan to ookinete surface proteins

All three protein models (Q8IJ96, Q04620, and Q71TS7) demonstrate high structural confidence, favorable global quality scores, and clean stereochemistry (Table 3).

**Table 3:** Protein model validation parameters showing stereochemistry (PROCHECK).

Target protein (UniProt ID)	pLDDT score	QMEANDisCo score	MolProbity score	Clashscore	Cβ deviations (>0.25 Å)
Q8IJ96	87.16 (confident)	0.68 ± 0.06	1.13 (99%)	1.29	0.00%
Q04620	83.82 (confident)	0.60 ± 0.06	1.14 (99%)	0.92	0.50%
Q71TS7	84.21 (confident)	0.57 ± 0.06	1.44 (96%)	0.92	0.00%

All three structures achieved mean pLDDT scores well above the 70 threshold (ranging from 83.82 to 87.16), indicating reliable backbone modeling suitable for structural analyses. The QMEANDisCo global scores (0.57–0.68) confirm good structural agreement with experimental reference structures of similar size in the PDB. Following explicit hydrogen addition and flip optimization, all three models achieved top-tier MolProbity scores (ranking in the 96%−99% globally) with exceptionally low clash scores (<1.30).

The structure quality of the three ookinete surface proteins (Q8IJ96, Q04620, Q71TS7) was checked in Ramchandran plot (Figure 2).

**Figure 2:**
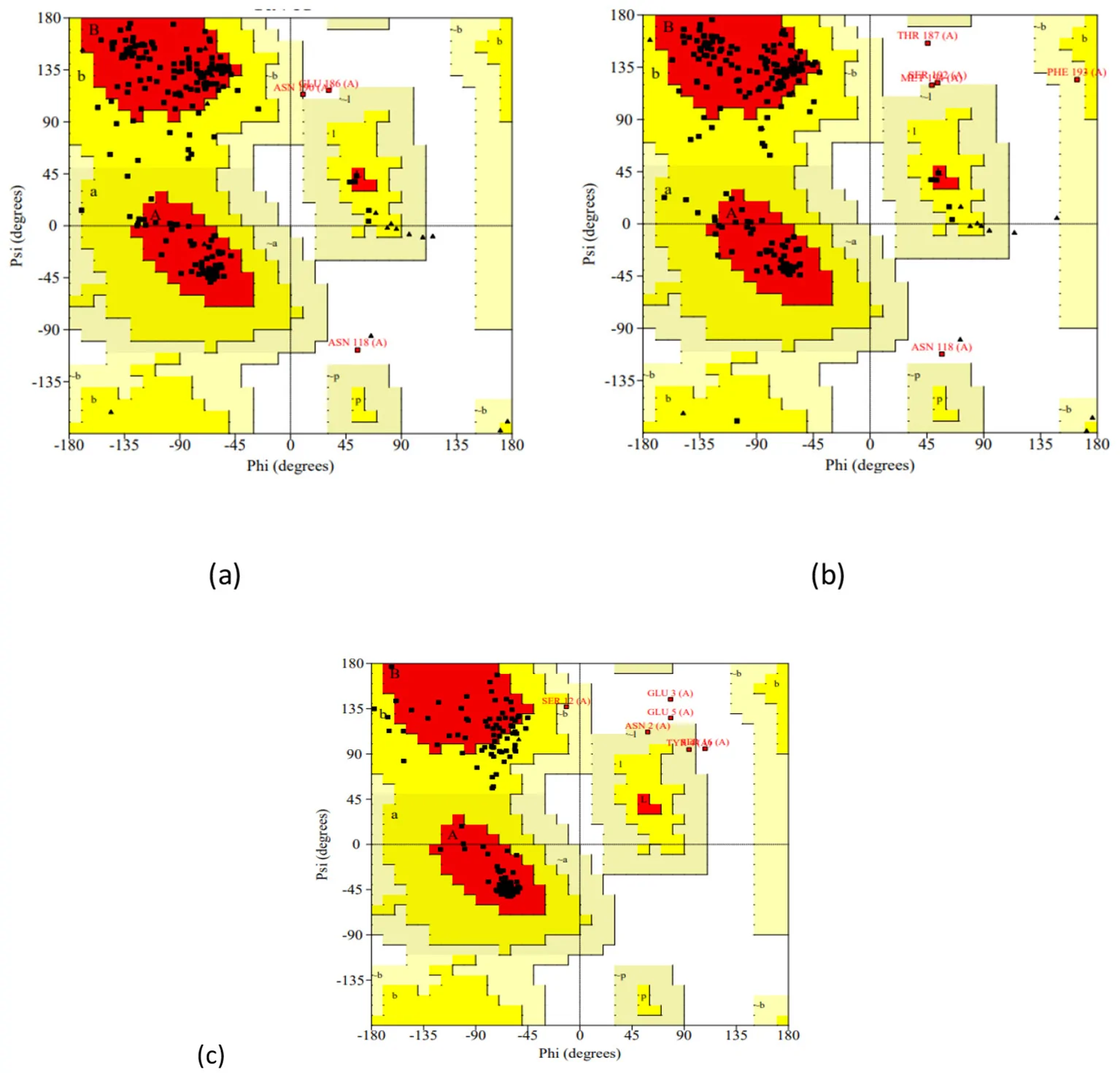
The Ramachandran plot calculated for three ookinete surface proteins; Q04620 and Q71TS7 **(a, b)** of *Plasmodium berghei* (Anka) and Q8IJ96 **(c)** of *Plasmodium falciparum* (3D7) computed by PROCHECK. The region marked by red color is the most favored region where residues fall. The two ookinete surface proteins of *Plasmodium berghei* (Anka) [Q04620 and Q71TS7] showed that more than 80% residues within favored regions (red) and another ookinete protein of *Plasmodium falciparum* (3D7), i.e., Q8IJ96, showed that more than 70% residues within favored regions (red).

To identify potential binding regions, triclosan was subjected to blind docking against three ookinete surface proteins (Q71TS7, Q04620, and Q8IJ96) (Figure 3). The predicted ligand–protein interactions were evaluated using the Glide XP docking score. Triclosan showed docking scores of −4.467 kcal/mol for Q71TS7-F1-model_v4, −4.645 kcal/mol for Q04620-F1-model_v4, and −5.936 kcal/mol for Q8IJ96-F1-model_v4. Among the three protein–ligand complexes, Q8IJ96-F1-model_v4 showed the most favorable docking score. The identified regions may therefore represent potential binding sites for triclosan and were subsequently considered for molecular dynamics analysis. The protein ligand interactions with the ligand triclosan revealed significant hydrophobic interactions for AF-Q8IJ96-F1-model_v4 with protein residues like Asp A125, Lys A21, Val A124, Ser A129, Glu A55, Ile A123, Glu A43, Tyr A42, His A41, Arg A23, Pro A126, Asn A20. Hydrogen bonding is involved in interactions with the residues like Ala A22, Tyr A77&Val A24; in the Q04620-F1-model_v4 hydrophobic interactions could be identified with protein residues like Tyr A77 to Ile A106, Cys A107, Ile A123, Lys A108 & Pro A109. Hydrogen bonding is involved in interactions with the residues like Lys A121; in the AF-Q71TS7-F1-model_v4 significant hydrophobic interactions with protein residues like Lys A23, Hie A41, Ile A123& Tyr A24 could be identified. Hydrogen bonding is involved in interactions with the residues like Lys A121, Ile A24, Leu A124, Glu A55 (Figure 4).

**Figure 3:**
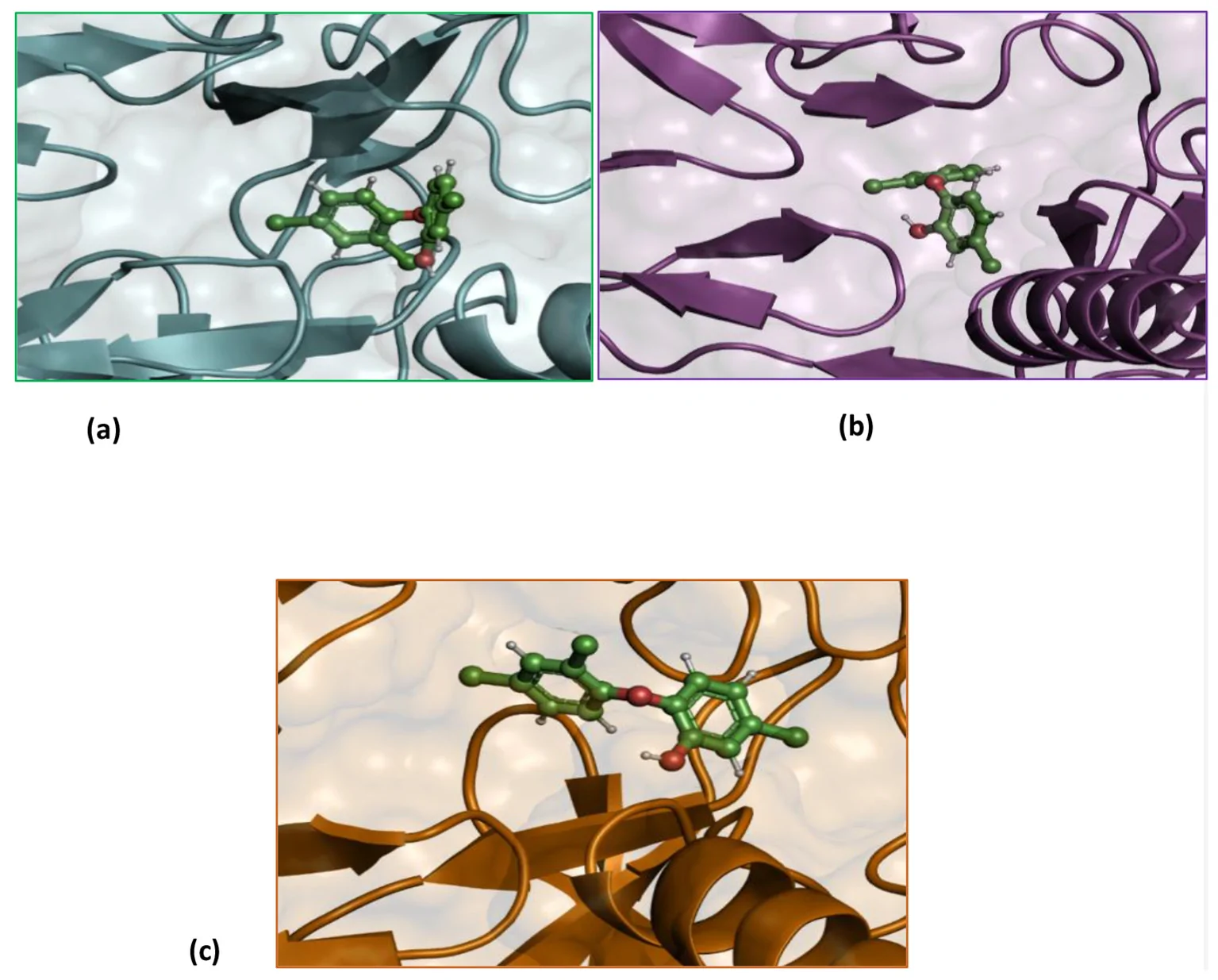
The ligands were found to be well docked in the binding pockets of **(a)** AF-Q8IJ96-F1-model_v4, **(b)** AF-Q04620-F1-model_v4, and **(c)** AF-Q71TS7-F1-model_v4 protein. The binding site is shown as the macromodel surface represented in cyan, purple, and brown color. The interaction of the binding residues of protein with ligands are shown in 2D form.

**Figure 4:**
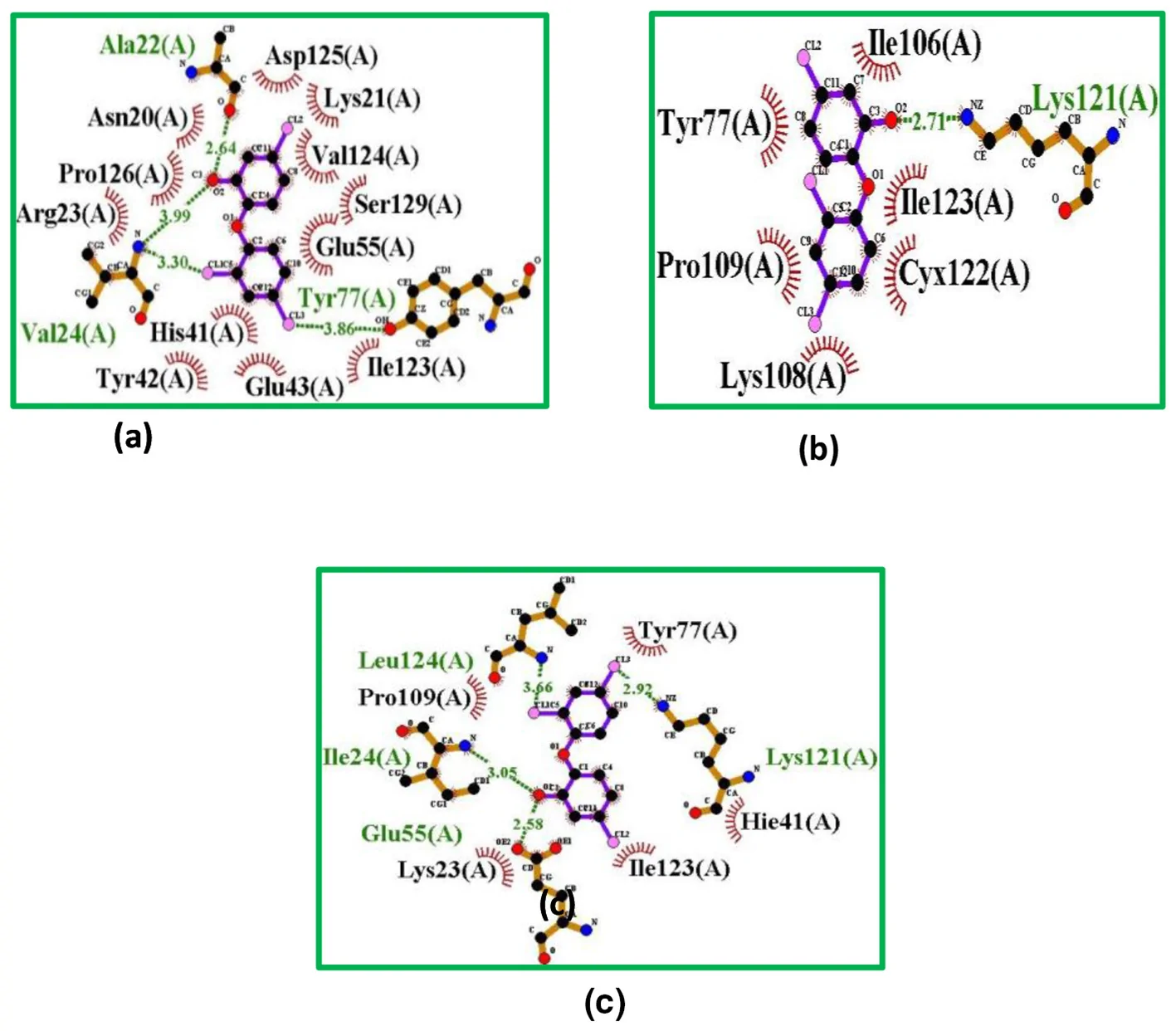
The interaction of the binding residues of **(a)** AF-Q8IJ96-F1-model_v4, **(b)** AF-Q04620-F1-model_v4, **(c)** AF-Q71TS7-F1-model_v4 protein with ligands are shown 2D form. Hydrogen bonds are represented by dashed lines and their lengths are denoted by numbers. Radial spokes arcs are the hydrophobic interactions. Residues that are 4 Å apart are shown only.

The structural stability of the three protein–ligand complexes was evaluated by monitoring the Cα RMSD of the proteins and the ligand RMSD over the 100 ns molecular dynamics trajectories. The RMSD profiles showed distinct conformational behavior among the three complexes (Figure 5). For the Q8IJ96–ligand complex from *P. falciparum*, the protein Cα RMSD exhibited an overall mean value of approximately 6.91 Å, with fluctuations between approximately 3.06 and 7.96 Å (Table 4). During the final 20 ns, the protein RMSD remained around 7.11 Å, indicating that the system approached a relatively stable conformational region toward the end of the simulation. The corresponding ligand RMSD averaged approximately 4.50 Å, with a mean value of approximately 4.53 Å during the final 20 ns, suggesting that the ligand remained associated with the binding region while undergoing conformational fluctuations. For the Q04620–ligand complex from *P. berghei*, the protein Cα RMSD showed a mean value of approximately 6.04 ± 0.99 Å, with fluctuations ranging from 3.66 to 10.75 Å. The average protein RMSD during the final 20 ns was approximately 5.93 Å. The ligand RMSD showed a mean value of approximately 5.02 ± 1.11 Å over the trajectory and decreased to approximately 4.08 Å during the final 20 ns, suggesting comparatively greater stabilization of the ligand toward the later stage of the simulation. The Q71TS7–ligand complex from *P. berghei* displayed the highest protein Cα RMSD, with an average value of approximately 8.01 ± 1.15 Å and a range of 4.15–10.52 Å. The protein RMSD increased toward the latter part of the simulation, reaching an average of approximately 9.10 Å during the final 20 ns, indicating greater conformational flexibility or structural rearrangement compared with the other two systems. In contrast, the ligand RMSD remained considerably lower, with an overall mean of approximately 2.06 ± 0.79 Å and an average of approximately 2.92 Å during the final 20 ns. This relatively lower ligand RMSD suggests that the ligand maintained a comparatively stable orientation within the binding pocket despite the larger protein conformational fluctuations (Table 4). We also acknowledge that RMSD alone is insufficient to establish binding affinity, and therefore the observed fluctuations should not be interpreted as direct evidence of weaker binding.

**Figure 5:**
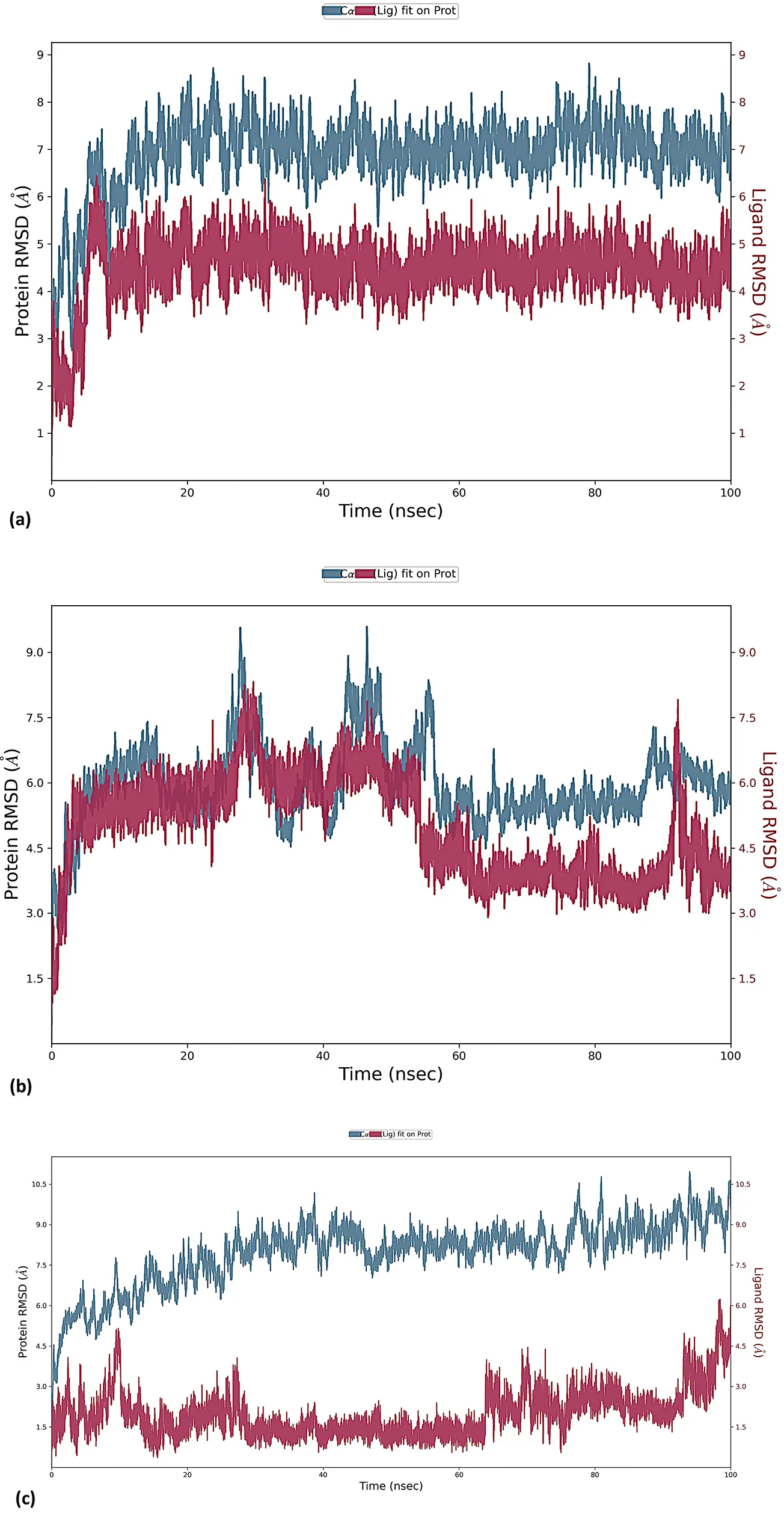
The plots illustrate protein RMSD [**(left)** *Y*-axis] and ligand RMSD [**(right)** *Y*-axis], with the latter indicating ligand stability within the protein binding pocket. **(a)** Q8IJ96 (*P. falciparum*), **(b)** Q04620 (*P. berghei*), and **(c)** Q71TS7 (*P. berghei*).

**Table 4:** Root mean square deviation (RMSD) values of the three-protein complex with triclosan.

Complex	Protein RMSD Å (mean ±SD)	Ligand RMSD Å (mean ±SD)
Q8IJ96-complex	6.91 ± 0.70	4.50 ± 0.54
Q04620-complex	6.04 ± 0.99	5.02 ± 1.11
Q71TS7-complex	8.01 ± 1.15	2.06 ± 0.79

### Triclosan displays minimal intrinsic toxicity to adult *An. stephensi* upon contact exposure

We first determined the intrinsic toxicity of triclosan to adult female *An. stephensi* mosquitoes using topical application assay. We tested six different concentrations of triclosan in the range of 0.193 to 19.3 ng/mg mosquito in this bioassay. We observed that triclosan had minimal effect on mosquito survival at all tested dosages. At the end of 24 h maximum mortality of only 6.2% was observed with the highest dosage 19.3 ng/mg mosquito. The minimum mortality was 4% at the lowest concentration of 0.193 ng/mg mosquito (Figure 6). Thus, triclosan had minimal toxicity when used topically. Acetone alone was included as the negative control, and no significant differences were observed between the acetone and untreated control groups, indicating that the observed effects were attributable to triclosan rather than the solvent.

**Figure 6:**
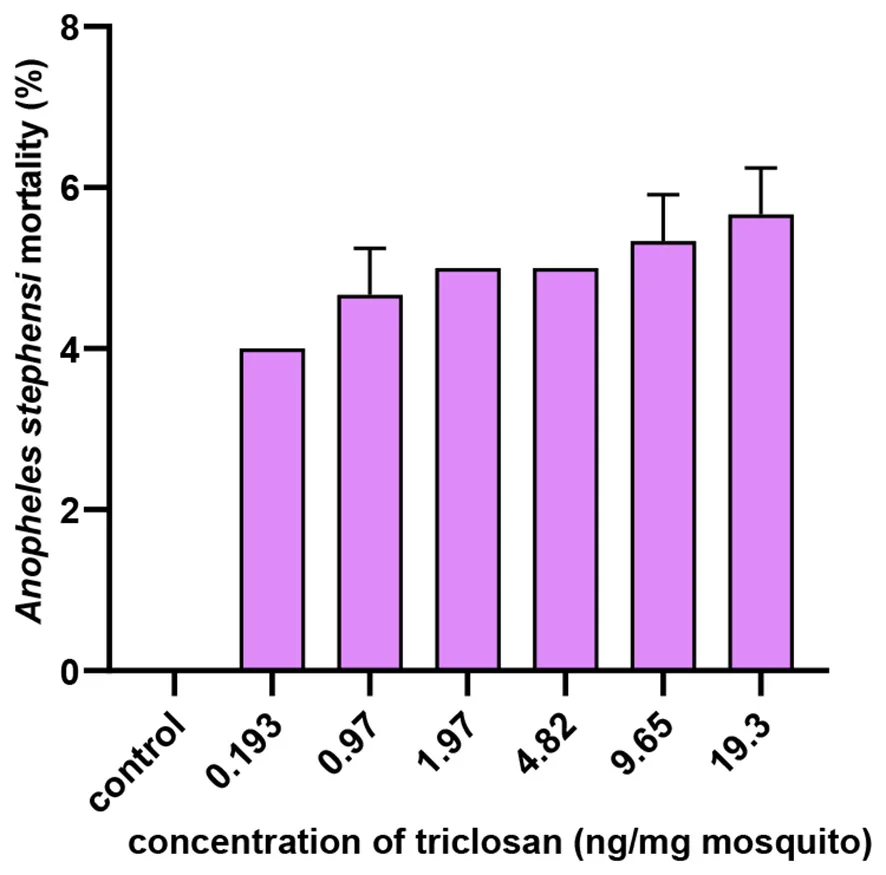
Female *An. stephensi* were topically treated on the thoracic pronotum with acetone or triclosan at different concentrations. Mortality was recorded after 24 h, and data are shown as mean ± SD.

We further tested six different concentrations of triclosan in the range of 2.89 to 289.54 ug/bottle on *An. stephensi* mosquitoes using bottle bioassay. Mosquito mortality ranged from 2%−4.6% at the tested dosages of triclosan indicating minimal toxicity of the drug on mosquito survival (Figure 7).

**Figure 7:**
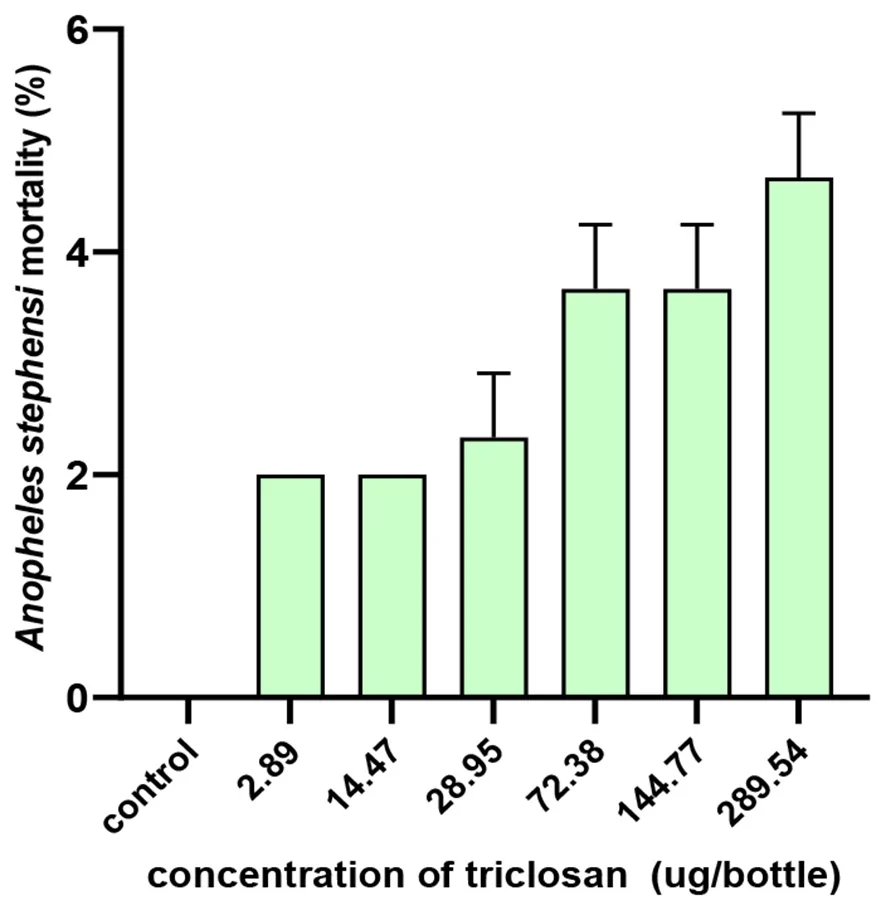
Female *An. stephensi* were exposed for 1 h (bottle bioassay) to acetone and graded concentrations of triclosan in coated glass bottles, and mortality after 24 h is shown as mean ± SD.

### Triclosan induces larval and adult death with oral exposure

We further examined the oral toxicity of triclosan in both larval and adult stages. The larvicidal potential of triclosan was determined at three concentrations viz., 10, 100, and 1,000 ppm against the third-instar larvae of *An. stephensi*. Among the three concentrations of triclosan, 100 and 1,000 ppm were significantly potent larvicides with near-complete mortality observed within 24 h of exposure. The 10 ppm concentration induced a delayed onset of mortality, with 100% mortality rate observed only after 48 h of exposure. Further, the 10 ppm dosage impaired the motility of exposed larvae during the first 24 h of exposure (Figure 8a).

**Figure 8:**
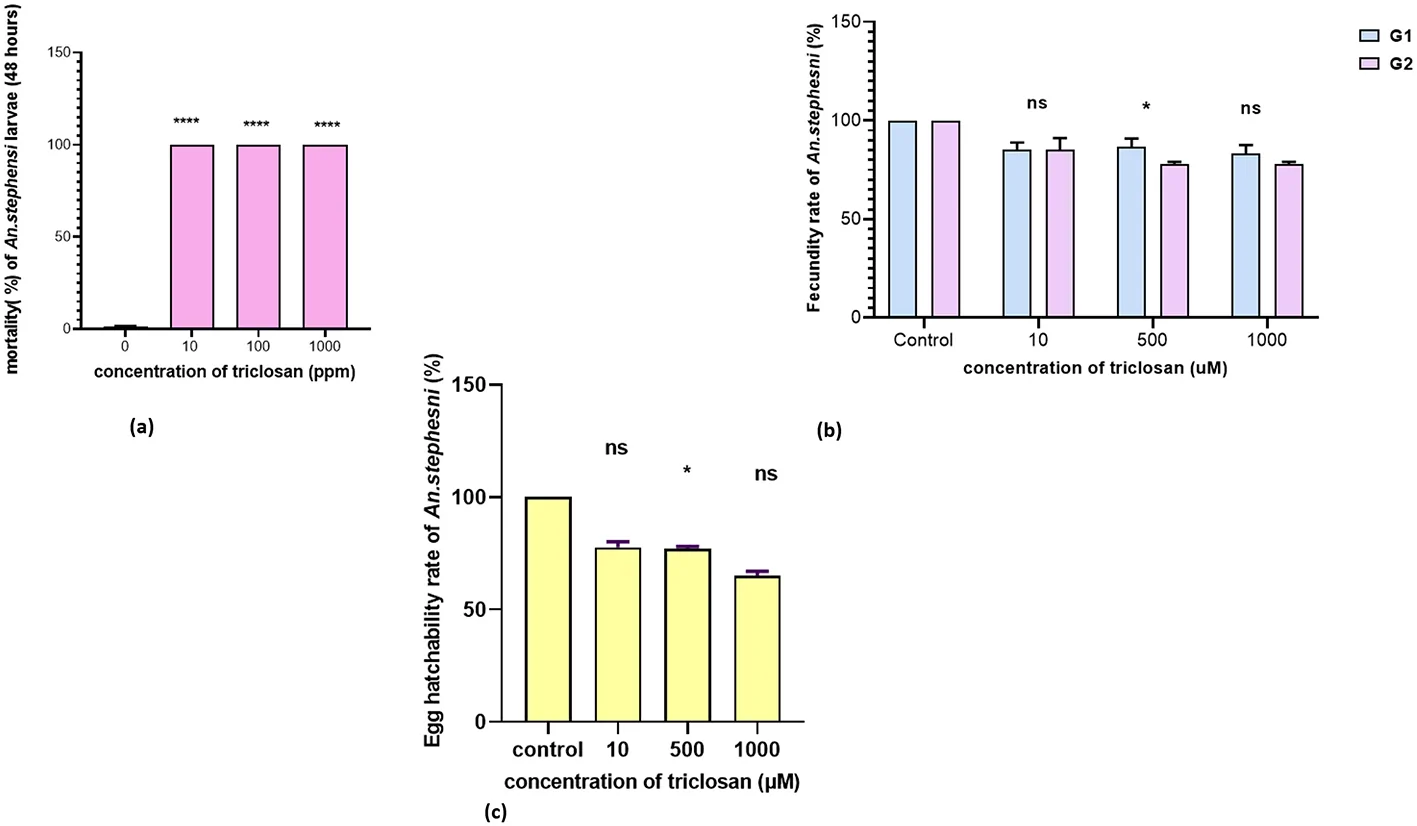
Effect of oral exposure on larval fecundity and hatchability. **(a)** Late 3 stage larvae of *An. stephensi* were exposed to three different concentrations of triclosan (10, 100, 1,000 ppm) and mortality was checked after 48 h; mean ± SD, *p value = 0.0419; ****p value < 0.0001. **(b)** Fecundity was checked for the gonotrophic cycle 1 and 2 at three different concentrations (10, 500, and 1,000 μM). Ns, non-significant. Data represents the three replicates of each test and mean ± SD. **(c)** Female *An. stephensi* were exposed to different concentrations (10, 500, and 1,000 μM) of triclosan (TSB) and bloodmeal given for egg development; fecundity with respect to control.

In a separate experiment, adult female *An. stephensi* were orally exposed to triclosan at different concentrations via a toxic sugar bait (TSB). Triclosan administration led to a significant, concentration-dependent reduction in mosquito survival across all tested doses (10, 50, 100, 250, 500, 1,000, and 2,000 μM). In the highest dose group (2,000 μM), more than 50% mortality was observed by day 8. For the intermediate concentration (500 μM), 50% mortality was reached on day 10, whereas at 100 μM, over 50% of mosquitoes remained alive beyond day 13.

Relative to control where mosquito survival was observed till day 25, 100% mortality was observed by day 12 in 2,000 μM concentration of triclosan as TSB. In the 500 μM and 1,000 μM triclosan-TSB groups mortality reached 100% by day 15 and day 14, respectively (Figure 9).

**Figure 9:**
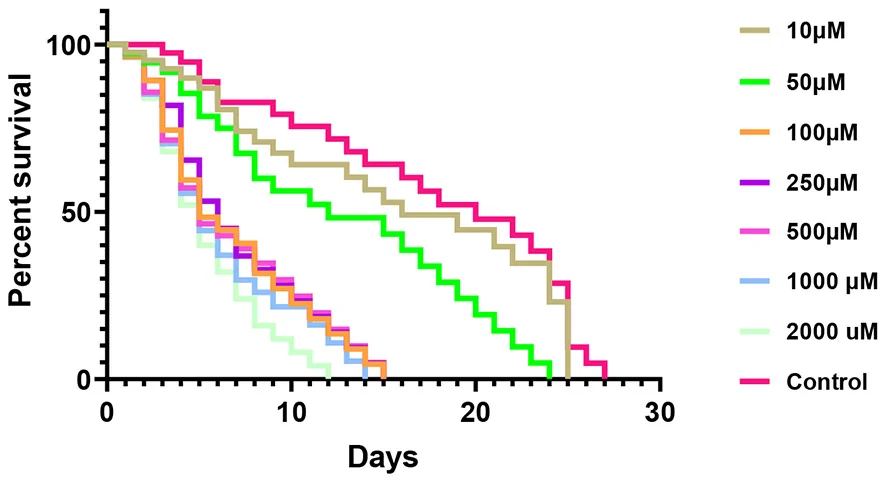
Feeding experiments of *Anopheles stephensi* with triclosan. Female *Anopheles stephensi* were orally fed seven different concentrations of triclosan (10, 50, 100, 250, 500, 1,000, 2,000) and vehicle control mixed with 5% sucrose. Mortality was recorded on a daily basis. Kaplan Meier curves displaying trend of mosquito survival with triclosan-TSB.

### Oral triclosan does not significantly affect fecundity but reduces egg hatchability at high concentrations

Oral feeding of triclosan did not affect the fecundity of exposed mosquitoes. There was insignificant reduction in the egg laying capacity of *Anopheles stephensi* mosquitoes with respect to control (untreated) upon oral feeding of different concentrations of triclosan across two gonotrophic cycles. Thus, no significant differences in fecundity were recorded across the two gonotrophic cycles G1 and G2 (Figure 8b). However, the statistical analysis for egg hatchability in mosquitoes with respect to control showed significant reduction at 500 μM (Figure 8c).

### Contact exposure to triclosan exposure does not significantly impair reproductive performance of *Anopheles stephensi*

Exposure to triclosan through the bottle bioassay did not significantly affect the reproductive performance of *Anopheles stephensi*. The control group showed approximately 100% fecundity, whereas mosquitoes exposed to 4 mM triclosan showed a lower mean fecundity of approximately 80%; however, this difference was not statistically significant (Figure 10a). Similarly, egg hatchability showed a numerical reduction from approximately 100% in the control group to 70%−75% following triclosan exposure, although this difference was also not statistically significant (Figure 10b). Thus, triclosan exposure at 4 mM showed a non-significant trend toward reduced fecundity and egg hatchability under the experimental conditions tested.

**Figure 10:**
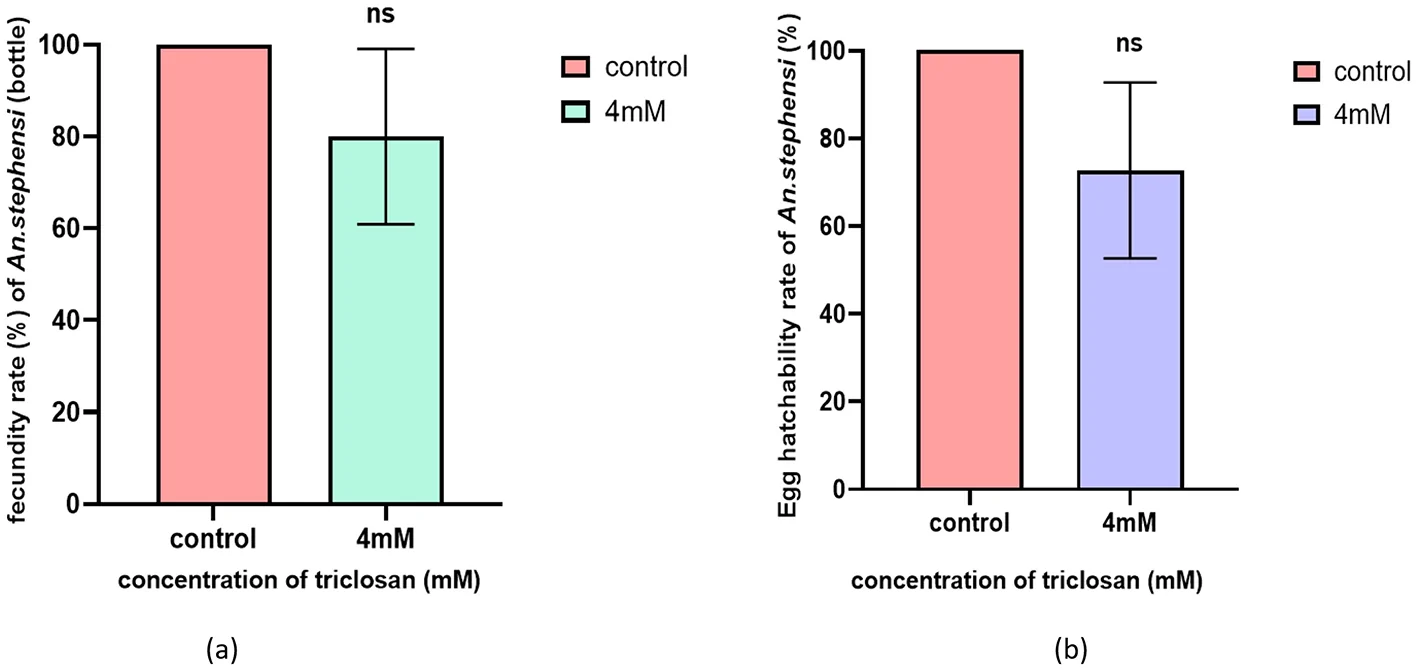
Effect of triclosan through contact exposure on reproductive performance of *Anopheles stephensi*. **(a)** Fecundity rate (%) and **(b)** egg hatchability rate (%) of *Anopheles stephensi* exposed to 4 mM triclosan compared with untreated control. Bars represent mean ± SD, “ns” indicates no statistically significant difference (*p* > 0.05).

### Triclosan suppresses *P. berghei* gametocyte exflagellation and ookinete formation *in vitro*

To determine the effect of triclosan on the sexual stages of *Plasmodium*, we studied *in vitro* male gametocyte exflagellation in the presence of triclosan at varying concentrations of 10, 50, and 70 μM. Interestingly, at concentrations 10 and 50 μM, there were no noticeable differences in the number of exflagellation centers compared to the control group. However, at 70 μM concentration, a significant reduction of 87% in exflagellation centers was observed. Specifically, the average number of centers decreased substantially from 6 to 0.75, indicating a potent inhibitory effect of triclosan at concentrations greater than 50 μM (Figure 11).

**Figure 11:**
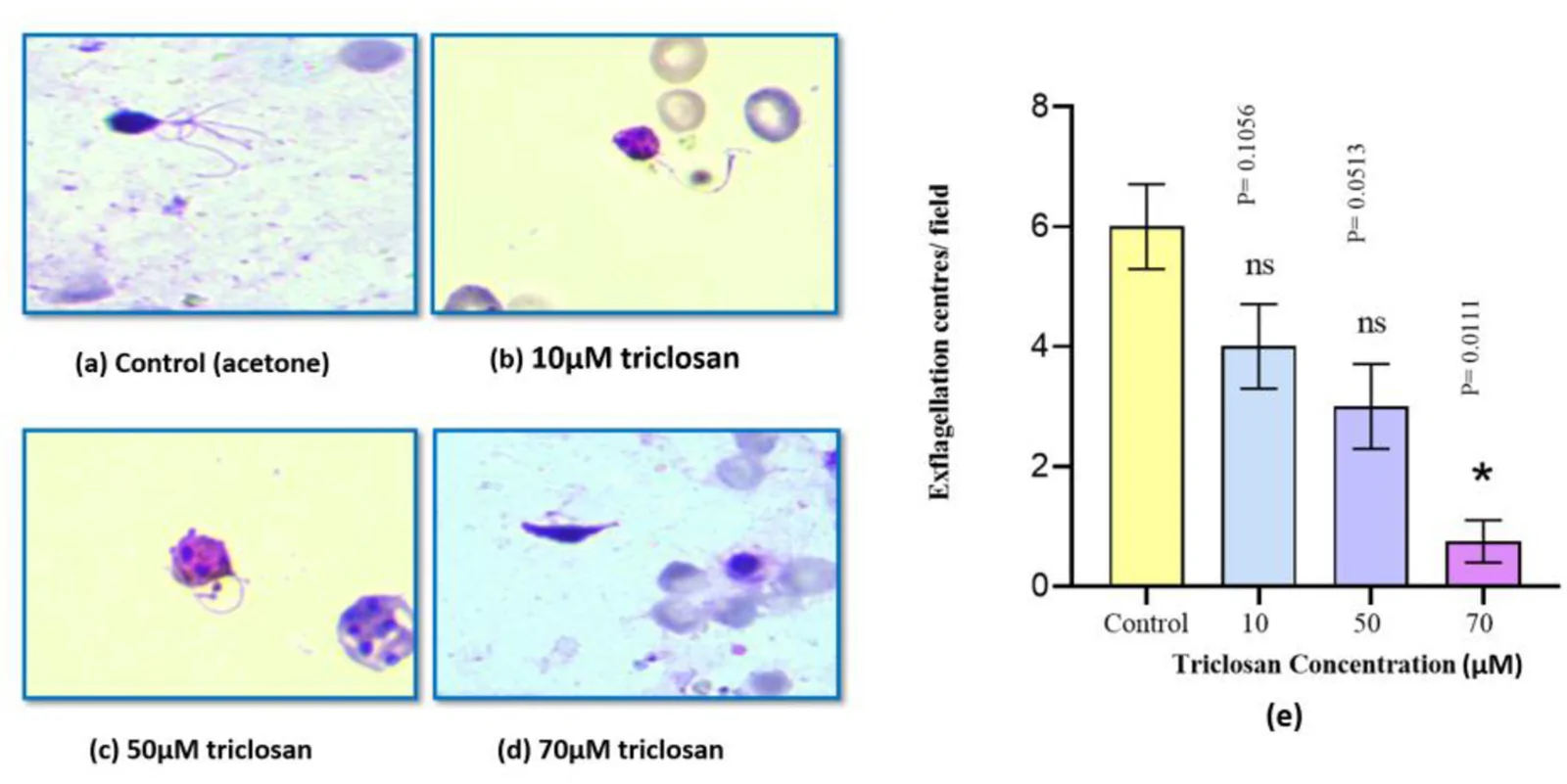
Micrographs taken at 40 × resolution showing *in vitro* exflagellation of *P. berghei* gametocytes in the presence of triclosan **(a)** control (acetone as a vehicle), **(b)** 10 μM triclosan, **(c)** 50 μM triclosan, **(d)** 70 μM triclosan, **(e)** Exflagellation centers/field across different treatment groups; shown as mean ± SD, ns, not significant, *p < 0.05.

We also examined the impact of triclosan on ookinete formation, a crucial stage for initiating the infectious sexual phase of the parasite's life cycle. Here also, triclosan was assessed at 10, 50, and 70 μM. At 10 μM there were no discernible difference observed in the number or morphology of ookinetes compared to the control group. However, at higher concentrations of 50 μM and 70 μM, significant reductions in both the quantity and morphology of ookinetes were noted. Specifically, at 50 μM, there was a substantial reduction of 97.7%, while at 70 μM, the reduction reached 99% (Figure 12).

**Figure 12:**
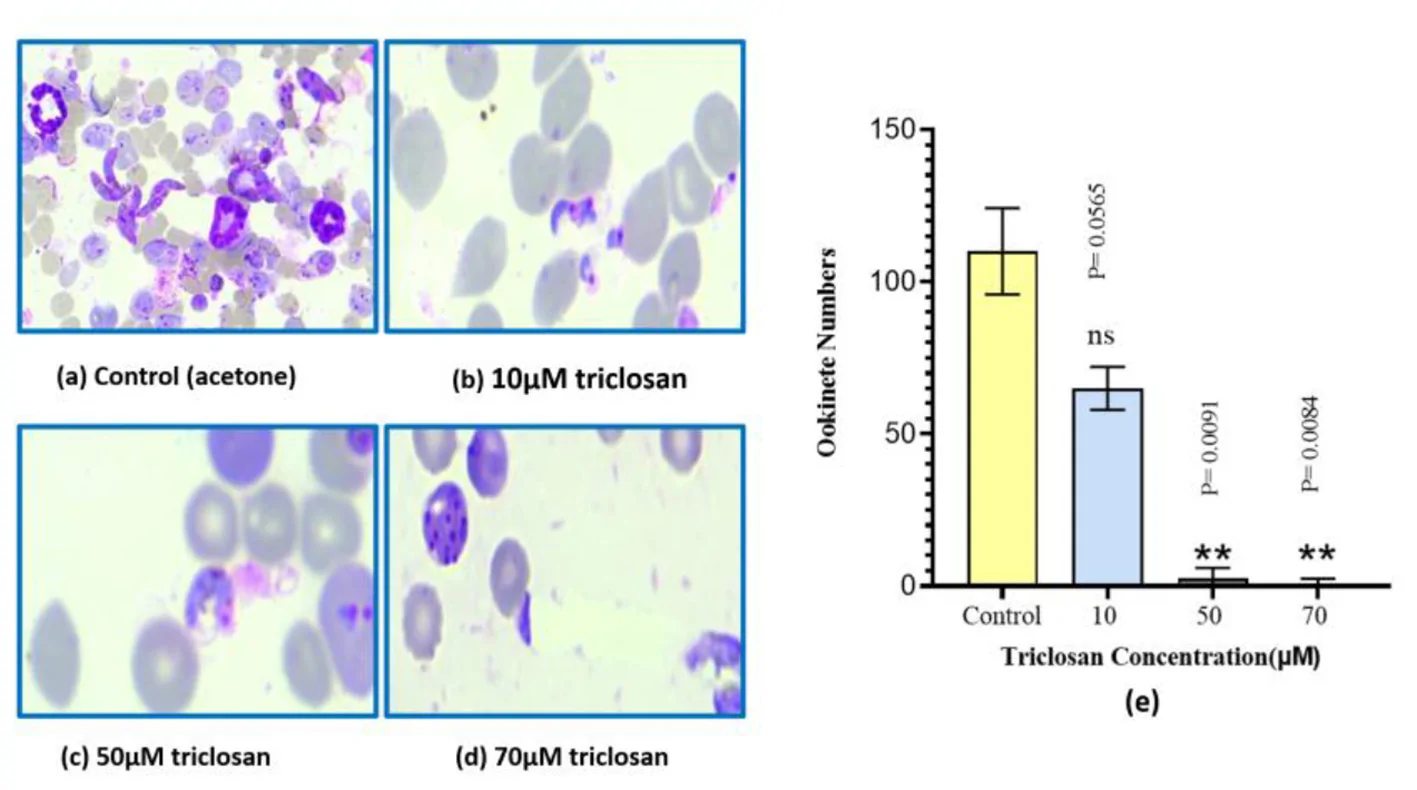
Micrographs taken at 40 × resolution showing *P. berghei* ookinete formation in the presence of triclosan **(a)** control (acetone as a vehicle), **(b)** 10 μM triclosan, **(c)** 50 μM triclosan, **(d)** 70 μM triclosan, **(e)** Ookinete numbers represented as mean ± SD for different treatment groups; ns, non-significant; **p < 0.01.

### Triclosan effectively blocks the *in vivo* maturation of *Plasmodium berghei* oocysts within the mosquito

We next investigated the potential of triclosan to inhibit sexual stage development of *Plasmodium berghei* in mosquito midgut upon contact-dependent exposure. We primarily studied the formation of berghei oocysts in mosquitoes which had been exposed to triclosan coated surfaces and then challenged with *P. berghei*. Here, we employed atovaquone (ATQ), a known contact-based inhibitor of oocyst development, as a standard control. ATQ was used at a concentration of 1 mM that resulted in 96.2% reduction in oocyst intensity in control mosquitoes. Subsequently, our experimental compound, triclosan, was tested at three different concentrations: 50, 100, 250, 500, 1,000, 2,000, and 4,000 μM. Encouragingly, we observed significant reductions in oocyst numbers with increasing concentrations of triclosan. Specifically, at 100 μM, there was a reduction of 45.7%, while at 1,000 μM reductions of 77.21% and at 2,000 and 4,000 μM reduction of 86.34% and 86.50%, respectively, were noted (Figures 13, 14).

**Figure 13:**
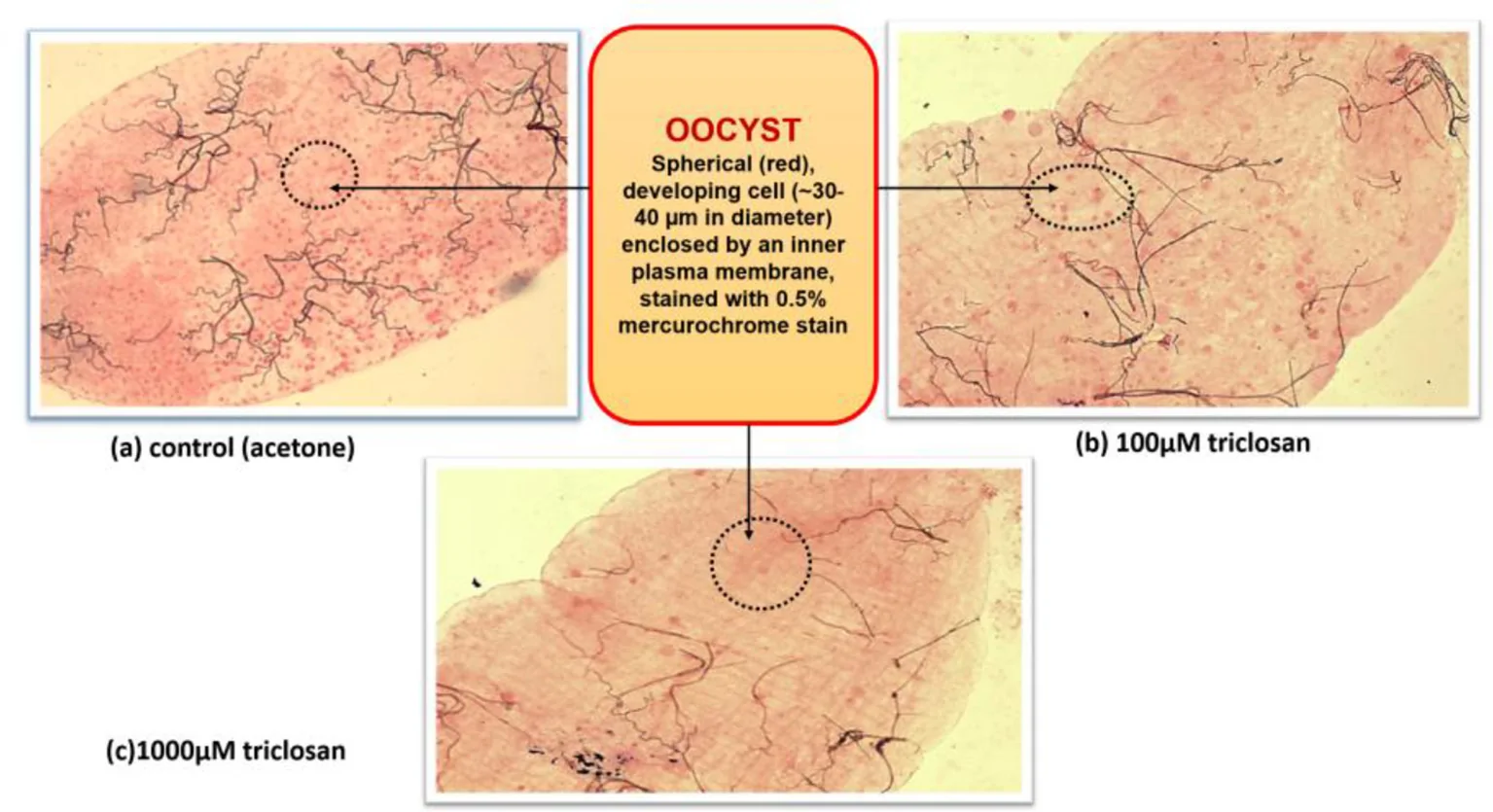
Midgut micrographs of *Anopheles stephensi* infected with *Plasmodium berghei* at 8–10 days post-infectious blood meal are shown for **(a)** vehicle control (acetone), **(b)** 100 μM triclosan, and **(c)** 1,000 μM triclosan. Images were captured at 40× magnification, and oocysts appear as red spherical structures in the midgut.

**Figure 14:**
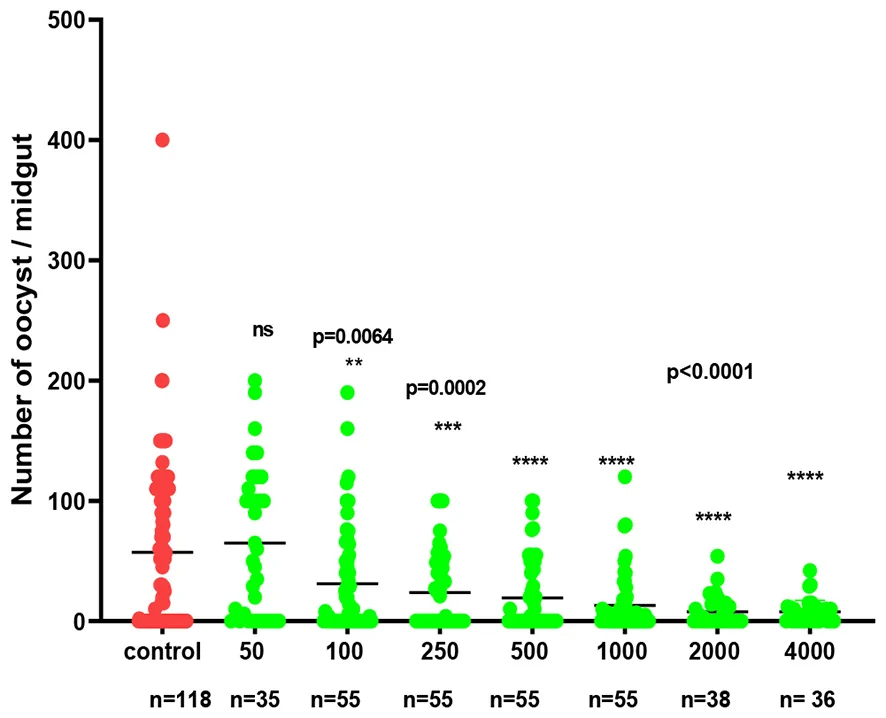
Following an infectious blood meal, *Anopheles stephensi* mosquitoes were subjected to bottle bioassays with triclosan at concentrations ranging from 50 to 4,000 μM for 60 min. Midguts were dissected from control and treated mosquitoes 8–10 days post-infection, stained with mercurochrome, and examined for oocyst presence. Transmission-blocking efficacy is presented as *Plasmodium berghei* oocyst intensity (oocysts per midgut) across experimental groups. Mean values are shown. ns, not significant; **p = 0.01; ***p = 0.001; ****p < 0.0001.

A dose–response curve for triclosan contact exposure was plotted using the above data. The ED_50_ value, obtained by interpolation, was 213.6 μM per bottle, corresponding to a surface concentration of 8.74 μM/m^2^ (95% CI: 112.3–380.1 μM per bottle; Figure 15).

**Figure 15:**
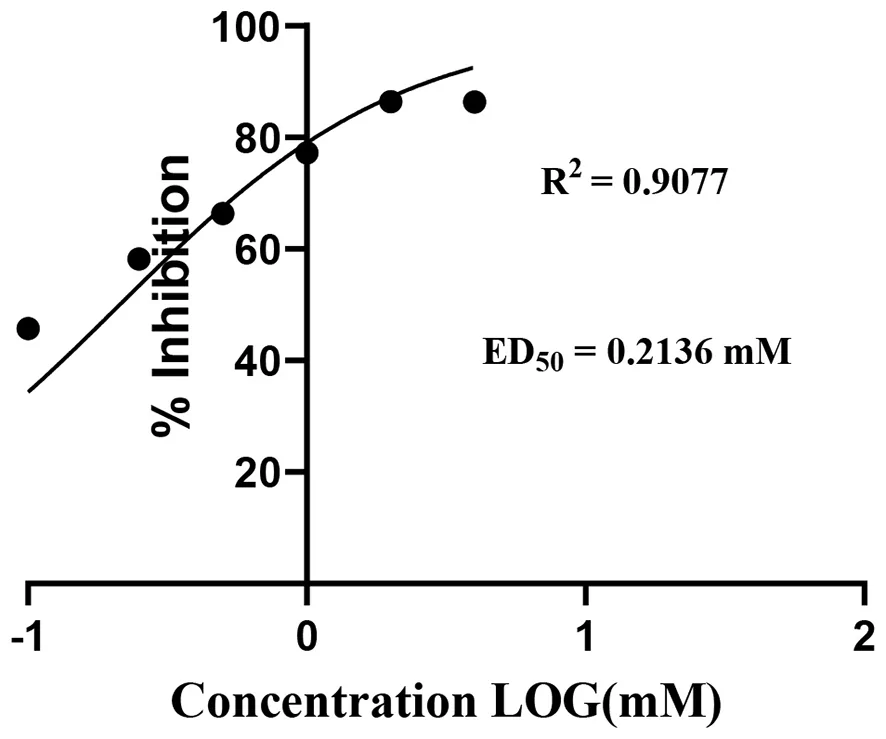
Dose–response curve fit for triclosan. The percentage inhibition of oocyst development (*Y*-axis) is plotted against the LOG concentration of triclosan (*X*-axis). The ED50 values were calculated by interpolation and are indicated in the graph. Values represent mean.

### Bioavailability of triclosan increased in a concentration-dependent manner

UPLC analysis confirmed the presence of triclosan in midgut tissues following exposure. Triclosan levels increased in a concentration-dependent manner. A statistically significant increase in midgut triclosan concentration was observed with increasing exposure concentration (Figure 16). One-way ANOVA revealed a significant difference among treatment groups (*p* < 0.05). *Post hoc* analysis indicated: significant increase in bioavailability at 2,000 μM compared to 100 μM (*p* = 0.0027). Highly significant increase at 4,000 μM compared to lower concentrations (*p* = 0.0007). These findings demonstrate dose-dependent accumulation of triclosan in mosquito midgut tissue.

**Figure 16:**
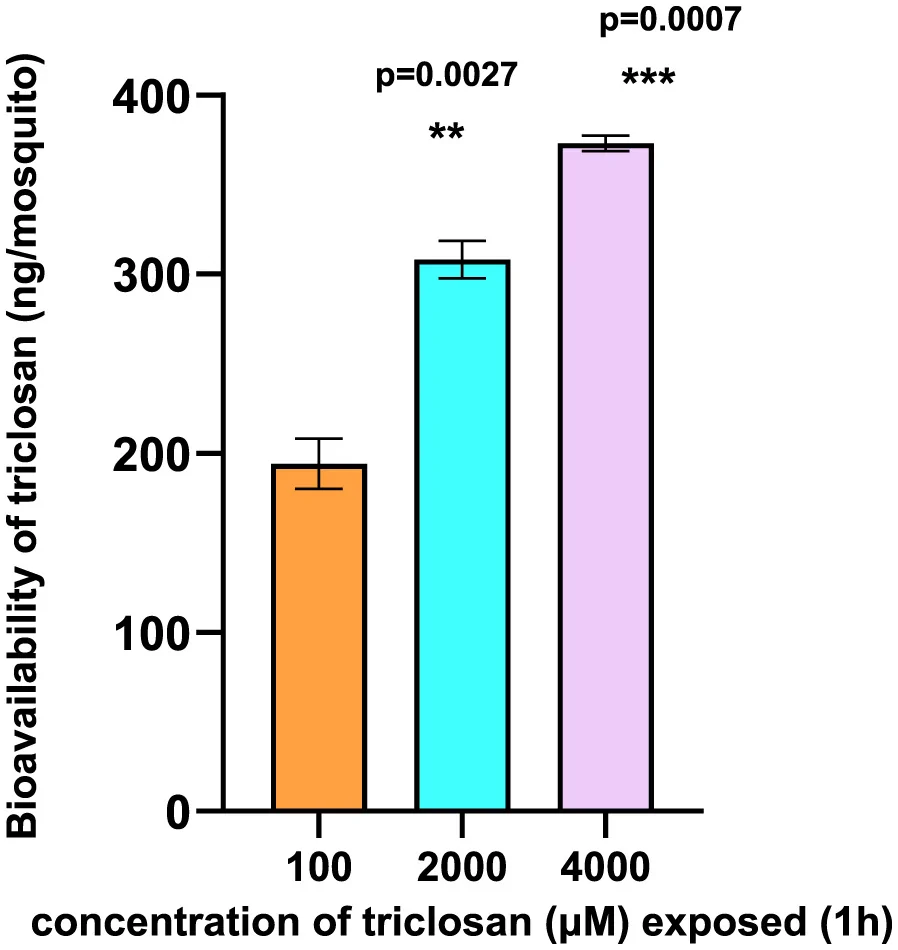
Bioavailability of triclosan in mosquitoes following acute exposure. Adult mosquitoes were exposed to varying concentrations of triclosan (100, 2,000, and 4,000 μM) for 1 h. The Y-axis represents the internal concentration (bioavailability) of triclosan measured in nanograms per mosquito (ng/mosquito). Asterisks indicate statistically significant differences among the triclosan treatment concentrations: **p < 0.01 and ***p < 0.001.

## Discussion

With mounting evidences on global insecticide resistance, it has become extremely critical to develop innovative strategies to tackle the rising threat of vector borne diseases. Blocking parasite transmission between human and vector is a promising strategy that may not only contain the pathogen but also bypass the problem of insecticide resistance. Triclosan is an antimicrobial molecule with well-documented antiparasitic activity against the asexual stages of *Plasmodium* (Surolia and Surolia, 2001). This study aimed to assess how triclosan influences the *in vivo* progression of the parasite's sexual stages. Based on the high lipophilicity of triclosan, we hypothesized that triclosan may be readily penetrate the mosquito cuticle (Thakur et al., 2025) and hinder with the sexual development of the parasite in the mosquito midgut. We initially performed *in silico* analysis to identify the possible interactions between triclosan and ookinete surface proteins of both *Plasmodium berghei* and *Plasmodium falciparum*. The molecular docking analysis provided a structural basis for the observed biological effects and suggested a potential interaction between triclosan and *Plasmodium* ookinete proteins. However, these findings are hypothesis-generating and may not provide the direct molecular mechanism for the observed inhibition. Further experimental validation, including biochemical, genetic, or functional studies, is required to confirm the molecular mechanism. Accordingly, the docking results are interpreted in the present study as supportive evidence rather than definitive proof of mechanism. Molecular docking revealed effective binding of triclosan to several critical *Plasmodium* ookinete surface proteins, evident from strong interaction and high binding affinity, with target proteins. The protein-ligand interactions involved both hydrophobic and hydrogen bonding.

Next, we determined the effect of triclosan on survival both through contact and oral modes of exposure. While contact exposure at different concentrations in topical application and bottle bioassays did not have deleterious impacts on the survival of *An. stephensi*, oral feeding negatively impacted the longevity and fecundity in adult mosquitoes. Larval feeding of triclosan resulted in significant mortality at higher tested concentrations of 100 and 1,000 ppm. The evidences on larval toxicity of triclosan are in line with a previous report by Xue and Qualls (2013). Xue and Qualls tested the larvicidal potential of triclosan both in purified form and as an active ingredient of disinfectant soaps on the late third or fourth instar larvae of *Culex quinquefasciatus* and observed 59% mortality with triclosan at an application rate of 0.1 g. In another study by Mdoe et al., the larvicidal potential of triclosan as part of disinfectant soaps has been elucidated in *An. gambiae* (Mdoe et al., 2014). Our findings on the larvicidal potential of triclosan corroborate with previous studies and ours is also the first report on the larvicidal potential of triclosan in *Anopheline* vectors. At a low concentration of 10 ppm, the toxicity of triclosan initially manifested as impaired motility within the first 24 h of exposure, followed by complete larval mortality by 48 h. Direct evidences in this context are lacking for mosquito vectors but in *Drosophila melanogaster*, triclosan exposure at concentrations as low as 0.1 mg/ml is shown to dramatically affect larval crawling (Zhang et al., 2021). We observed similar motility impairments in L3 of *stephensi* at low concentrations of triclosan. As regards fecundity, oral triclosan displayed reproductive toxicity with significant reduction in the reproductive output which was concentration dependent. This is in line with the negative impact of triclosan on fertility in *D. melanogaster* that was also dose-dependent and adversely affected the ovarian tissue (Zhang et al., 2021).

However, for the exflagellation assays, ATQ could not be employed as a control, as ATQ does not inhibit *Plasmodium berghei* microgametocyte exflagellation even at concentrations up to 300 μM, indicating minimal direct activity against male gamete activation (Fowler et al., 1995). ATQ on the other hand, is demonstrated to be a potent inhibitor of post-fertilization parasite development, with partial inhibition of ookinete formation at 0.05–0.1 μM and nearly complete inhibition at 0.25 μM (Fowler et al., 1995).

Next, mosquitoes were exposed to triclosan-coated bottles and subsequently infected with *P. berghei* 12 h after drug exposure. Interestingly, contact exposure to triclosan significantly reduced oocyst intensity in a clear dose-dependent manner. At 100 μM, oocyst load decreased by 45.7%, whereas the highest concentration of 2,000 μM (2 mM) and 4,000 μM (4 mM) resulted in reductions (almost same) exceeding 86%. The calculated surface ED_50_ of triclosan as a contact toxin was 8.74 μM/m^2^. Although slightly higher, this value is comparable to the surface IC_50_ for parasite inhibition by ATQ (1.77 μM/m^2^) following a 60-min tarsal exposure (Paton et al., 2019), suggesting that triclosan exerts biologically relevant antiparasitic activity upon contact. The concentration-dependent increase in triclosan levels within the midgut confirms effective uptake and retention following short-term exposure. Furthermore, differences in their respective modes of action may underlie the observed variation in the inhibitory concentrations. Mosquitoes were exposed 12 h after the infectious blood meal to model a realistic post-blood-meal contact exposure scenario and to follow an experimental strategy previously used to demonstrate post-infection transmission blocking by contact-delivered antimalarial compounds (Paton et al., 2019). At this time point, *Plasmodium* undergoes important developmental transitions within the mosquito midgut. Because exposure was initiated 12 h after the infectious blood meal, the experimental design is unlikely to capture effects on the earliest sexual stages of parasite development. The observed reduction in oocyst numbers therefore suggests that triclosan may interfere with subsequent parasite development leading to oocyst formation. However, the present experimental design does not establish the precise developmental stage at which triclosan exerts its effect. Therefore, future studies evaluating exposure at multiple time points before and after 12 h post-blood meal, together with stage-specific assessment of parasite development, will be required to identify the optimal intervention window and further define the stage-specific activity of triclosan.

These results are the first documented evidences on the inhibitory effects of triclosan on the sexual stages of malaria parasite particularly, gametocyte, ookinete and oocyst. The reduction in oocyst intensity is suggestive of two possible *in vivo* mechanisms which may underlie the observed effects of triclosan (i) inhibition of gametocyte exflagellation (ii) disruption of ookinete to oocyst transition. It appears that triclosan primarily targets the early stages of parasite's sexual development. However, triclosan may not be effective in the later stages of sexual development as indicated in a recent study by Farell et al., who did not observe inhibition of oocyst to sporozoite transition (Farrell et al., 2025).

Although our findings demonstrate that triclosan inhibits several mosquito-stage *Plasmodium* developmental phenotypes, including gametocyte exflagellation, ookinete formation, and oocyst development, the precise mechanism underlying these effects remains to be established. Further, Triclosan is a broad-spectrum antimicrobial compound and may therefore influence *Plasmodium* development indirectly through alterations in the mosquito midgut microbiota (Sinicropi et al., 2022). The mosquito microbiota is known to influence *Plasmodium* development (Romoli and Gendrin, 2018), and antimicrobial-mediated changes in microbial composition could also potentially contribute to the observed reduction in parasite development (Gendrin et al., 2015).

Alternatively, the observed inhibition could also reflect a broader antiplasmodial effect rather than selective targeting of a particular sexual stage.

Triclosan is a well-established inhibitor of enoyl-acyl carrier protein reductase (ENR/FabI), a key enzyme of the apicoplast fatty acid synthesis (FAS-II) pathway (Surolia and Surolia, 2001). Thus, inhibition of FabI represents a plausible alternative mechanism for the observed transmission-blocking activity. Although our docking analysis predicted potential binding to P25/P28 proteins, we cannot exclude the possibility that the observed effects are mediated through FabI inhibition, alterations in the mosquito midgut microbiome, or other mechanisms. The docking results are hypothesis-generating and do not establish a definitive molecular target. Future biochemical and experimental studies are required to distinguish between these possibilities.

Acetone was included as a solvent control, and no significant differences were observed between acetone-treated and untreated controls, suggesting that acetone alone was unlikely to account for the observed effects under the experimental conditions. Nevertheless, potential effects of the solvent on cuticular penetration or mosquito physiology cannot be completely excluded. Taken together, these findings support the transmission-blocking potential of triclosan but do not establish whether its effects are mediated by a direct action on the parasite, alterations in the mosquito microenvironment, or a combination of mechanisms. Future studies incorporating mosquito microbiome profiling, stage-specific parasite assays, and targeted validation of candidate molecular targets will be important for distinguishing these possibilities and defining the mechanism of triclosan-mediated transmission blocking.

Thus, in the present study, contact exposure to triclosan exhibited strong antiparasitic activity while imposing negligible toxicity or fitness costs on mosquitoes in terms of survival. Mortality did not exceed 5.6% across all tested concentrations in both bottle bioassays and topical application, indicating its selective activity against the parasite without significantly affecting mosquito viability. This property makes triclosan a unique candidate that could suppress parasite transmission without exerting strong selection pressure on mosquitoes that could drive resistance development. Additionally, triclosan showed no significant effect on mosquito fecundity or egg hatchability, suggesting limited disruption of population dynamics. This is beneficial because it reduces the risk of resistance, a common issue with insecticides (Kumar et al., 2024). These results are in line with the transmission blocking effects of two other anti-*plasmodium* drugs- atovaquone and sub-lethal doses of ivermectin (Jeena et al., 2025) which have shown similar low-toxicity, transmission-blocking effects in *Anopheles* mosquitoes, targeting the parasite while leaving the vector largely unharmed (Paton et al., 2019).

An important limitation of the present study is that parasite development was evaluated up to the oocyst stage and did not extend to sporozoite development or infectivity. Although reduction in oocyst burden supports the transmission-blocking potential of triclosan, successful inhibition of oocyst development does not by itself establish an effect on subsequent sporozoite production or transmission to the vertebrate host. Future studies should therefore evaluate sporozoite development, salivary gland invasion, and parasite infectivity to determine whether the observed effects on early mosquito-stage development translate into inhibition of the complete transmission cycle.

## Conclusion

These results highlight triclosan's potential not only as an anti-parasitic agent but also as a viable transmission-blocking strategy in malaria vector control. In conclusion, while triclosan has shown considerable promise in inhibiting *Plasmodium* transmission by targeting the early sexual stages of malaria parasite, further studies are required to investigate its precise mechanisms of action, long-term safety, and efficacy in field conditions. Nonetheless, these findings make triclosan a promising candidate for further exploration in malaria control strategies, particularly for interrupting parasite transmission within vector populations.

## Data Availability

The original contributions presented in the study are included in the article, further inquiries can be directed to the corresponding authors.
